# Prevalence of hypertension among patients aged 50 and older living with human immunodeficiency virus

**DOI:** 10.1097/MD.0000000000015024

**Published:** 2019-04-12

**Authors:** Patrick Dakum, Gbenga Ayodele Kayode, Alash’le Abimiku, Yohanna Kambai Avong, James Okuma, Ezenwa Onyemata, Taofeekat Ali, Victor Adekanmbi, Olalekan Uthman

**Affiliations:** a Institute of Human Virology; b International Research Centre of Excellence, Institute of Human Virology, Nigeria, Maina Court, Herbert Macaulay Way, Central Business District, Abuja, Nigeria; c Division of Population Medicine, School of Medicine, Cardiff University, Cardiff; d Warwick-Centre for Applied Health Research and Delivery, Division of Health Sciences, Warwick Medical School, The University of Warwick, Coventry; e International Health Group, Liverpool School of Tropical Medicine, Liverpool, Merseyside, UK.; f Julius Global Health, Julius Center for Health Sciences and Primary Care, University Medical Centre Utrecht, Utrecht, The Netherlands.; g Institute of Human Virology University of Maryland School of Medicine, Baltimore.

**Keywords:** elderly, HIV, hypertension prevalence

## Abstract

**Background:**

Hypertension is one of the common medical conditions observed among patients aged 50 years and elder living with HIV (EPLWH) and to date no systematic review has estimated its global prevalence.

**Purpose:**

To conduct a systematic review to estimate the global prevalence of hypertension among EPLWH.

**Data Sources:**

PubMed/MEDLINE, Embase, the Cochrane Library, and Global Health databases for relevant publications up till May 25, 2018.

**Study Selection:**

Observational studies (cohort or cross-sectional studies) that estimated the prevalence of hypertension among EPLWH.

**Data Extraction:**

Required data were extracted independently by three reviewers and the main outcome was hypertension prevalence among EPLWH.

**Data Synthesis:**

The 24 (n = 29,987) eligible studies included were conducted in North America, Europe, Africa, and Asia. A low level bias threat to the estimated hypertension prevalence rates was observed. The global prevalence of hypertension among EPLWH was estimated at 42.0% (95% CI 29.6%–55.4%), *I*^*2*^ = 100%. The subgroup analysis showed that North America has the highest prevalence of hypertension 50.2% (95% CI 29.2% –71.2%) followed by Europe 37.8% (95% CI 30.7%–45.7%) sub-Saharan Africa 31.9% (95% CI 18.5% –49.2%) and Asia 31.0% (95% CI 26.1%–36.3%). We found the mean age of the participants explaining a considerable part of variation in hypertension prevalence.

**Conclusion:**

This study demonstrated that two out of five EPLWH are hypertensive. North America appears to have the highest prevalence of hypertension followed by Europe, sub-Saharan Africa (SSA) and Asia respectively. Findings from this study can be utilized to integrate hypertension management to HIV management package. (Registration number: CRD42018103069)

## 1 Introduction

Following the advent of antiretroviral therapy (ART), survival of human immunodeficiency virus (HIV) infected patients has improved dramatically due to decline in progression of the disease to acquired immunodeficiency syndrome (AIDS) and HIV-related deaths.^[1–4]^ As ART coverage and retention of HIV infected patients on treatment increase, more HIV infected patients are attaining geriatric age.^[5]^ However, as life expectancy of HIV infected patients is improving, studies have revealed important health implications of HIV infection and ART among middle-aged and older adults. Previous studies have shown that HIV infection and use of ART can potentiate early onset of geriatric syndrome.^[6,7]^

In addition, it has been reported that this newly aging population of HIV infected patients are experiencing early onset of diseases that are seen in older population of people who are not infected with the virus. For example studies have revealed that HIV infected patients who are on ART were at higher risk of having HIV associated non-AIDS conditions (HANA) such as hepatic, renal, pulmonary, cancers, bone, neurological, metabolic and cardiovascular diseases.^[8–15]^ Of all these co-morbid conditions, cardiovascular disease is the most important cause of deaths among HANA conditions and its major underlying risk factor is hypertension.^[16]^ Cardiovascular disease occurs more and earlier in HIV-infected patients than HIV-uninfected patients because of their exposure to HIV and ART. It is believed that HIV-induced immune activation and/or ART-associated dyslipidemia facilitate early onset of cardiovascular disease in HIV-infected patients.^[17]^

With the emergence of cardiovascular diseases, it is clinically relevant to restructure HIV management to allow the integration of cardiovascular diseases management into HIV management package. It will be challenging to carry out this integration without having adequate information on the prevalence of hypertension among HIV-infected patients. Although few systematic review studies have been conducted to estimate the prevalence of hypertension among HIV-infected patients,^[18–20]^ none of these studies estimated the prevalence of hypertension among elderly with HIV despite the strong synergy between HIV and aging. Thus, it is the aim of this study to estimate the prevalence of hypertension among elderly people living with HIV (EPLWH) infection. This will serve as important information for HIV program planning and implementation.

## 2 Methods

The study protocol for this systematic review was developed based on the Preferred Reporting Items for Systematic Reviews and Meta-Analyses (PRISMA) guidelines.^[21]^ Detailed information on the study rationale and methods were pre-specified in the protocol registered in the International Prospective Register of Systematic Reviews (PROSPERO) with an identification number CRD42018103069.

### 2.1 Data sources and searches

We followed standard guidelines for integrating existing systematic reviews into new reviews.^[22,23]^ Existing systematic reviews on the prevalence of hypertension in people living with HIV were used as a starting point to identify relevant studies and searches were updated accordingly. We searched PubMed/MEDLINE, Embase, the Cochrane Library, and Global Health databases for relevant publications up till May 25, 2018, using keywords related to HIV and hypertension. In addition, abstract presentations in HIV/AIDS and infectious diseases conferences were searched. Also, reference lists of identified publications were checked for additional relevant articles. The search was performed without restriction based on geographic location and language. However, the update search was limited by year of publication (January 2015 to May 25, 2018).

### 2.2 Eligibility criteria

We evaluated each identified study against the following predefined selection criteria:

### 2.3 Participants/population

Elderly population, aged 50 years and above living with HIV/AIDS.

### 2.4 Exposure(s)

Treatment naïve or on any ART and whether on antihypertensive medications, statin or aspirin or not.

### 2.5 Comparator(s)

Not applicable.

### 2.6 Outcome

Essential hypertension (also called primary or idiopathic hypertension), defined as persistent systolic blood pressure (SBP) of ≥140 mm Hg and/or had diastolic blood pressure (DBP) ≥90mm Hg regardless of age and sex or hypertension deducible from the use of antihypertensive drugs or self-reported physician-diagnosed cases. We excluded studies that included subjects with pregnancy-induced hypertension, pre-eclampsia, malignant, portal, pulmonary, renal, intracranial, or ocular hypertension.

### 2.7 Study selection

Three authors (GAK, OAU, and PD) independently assessed the titles and abstracts of the publications found by our literature search based on the eligibility criteria that we pre-specified in the study protocol. All disagreements were resolved by evaluating the whole article. We only included observational studies that estimated the prevalence of hypertension among elderly people with HIV.

### 2.8 Data extraction and quality assessment

Data were extracted from all the selected publications by three authors independently (GAK, TA, and PD). Information on first author, study design, period of data collection, study location, use of ART, sample size, average age of the participants, prevalence of hypertension, and diagnosis of hypertension were obtained. All discrepancies during data extraction were resolved by consensus after discussion. We assessed the quality of the selected studies using the critical appraisal tool for evaluating the qualities of papers included in systematic reviews addressing questions of prevalence.^[24]^ The quality of articles included in the analysis were assessed for the risk of bias in 7 domains: representative sample, participants recruitment, adequate sample size, appropriate description of participants and setting, data completeness/nonresponse, unbiased outcome assessment, and appropriate statistical analysis. We reported risk of bias in each domain as low risk, high risk, or unclear.

### 2.9 Data synthesis and analysis

For the meta-analysis, we first stabilise the raw prevalence of estimate from each study using the logit transformation proportion suitable for pooling and then pooled the prevalence estimates using the DerSimonian–Laird random effects model.^[25]^ We performed leave-one-study-out sensitivity analysis to determine the stability of the results. This analysis evaluated the influence of individual studies by estimating the pooled hypertension prevalence in the absence of each study.^[26]^ We assessed the heterogeneity among studies by inspecting the forest plots and using the chi-squared test for heterogeneity with a 10% level of statistical significance, and using the *I*^*2*^ statistic where we interpreted a value of 50% as moderate heterogeneity.^[27,28]^ We assessed the possibility of publication bias by evaluating a funnel plot for asymmetry. Because graphical evaluation can be subjective, we also conducted Egger's regression asymmetry test^[29]^ as formal statistical test for publication bias.

Furthermore, we explored the effect of study-level factors on the overall pooled hypertension prevalence estimates using subgroup and meta-regression analyses: publication year, study design (cross-sectional or longitudinal study), study region (North America, Europe, Africa, or Asia), sample size, mean age, and percentage male. Univariable random-effects logistic regression analysis was conducted to investigate the impact of study-level factors (listed above) on the pooled hypertension prevalence. Univariable random-effects logistic regression analyses were used to investigate the bivariate relationship between each study-level factor and prevalence of hypertension estimates. Meta-analysis results were reported as combined hypertension prevalence with 95% confidence intervals (CIs), while meta-regression results were reported as odds ratio with 95% CIs. All *P*-values were exact and *P*-values <.05 were considered statistically significant. Analyses were conducted using Stata version 14 for Windows (Stata Corp, College Station, Texas). This systematic review was reported according to the PRISMA guideline.^[21,30]^

### 2.10 Role of the funding source

No funding was received to conduct this study

### 2.11 Ethical approval

We conducted a systematic review of published evidence; thus no ethical clearance was required.

## 3 Results

### 3.1 Study characteristics

The PRISMA flow diagram shows the results of the literature search carried-out to identify and select eligible literature published after the existing systematic reviews^[18,19]^ that were identified. In addition to the 12 eligible full-text articles that were identified from the existing systematic reviews, the results of the literature search yielded 560 citations. We identified 31 full-text articles for critical review after removing 529 articles due to duplication of articles and failure to meet the eligibility criteria. Of the 31 articles from the updated search that were critically appraised, only 12 articles were included in the analysis; indicating that a total of 24 articles were considered in the analysis. Twenty-four studies, involving 29,987 elderly people living with HIV met the inclusion criteria and were included in this meta-analysis. Characteristics of included studies are summarized in Table 1. The studies were published between 2009 and 2018. Most of the studies were from the United States of America (n = 10, 41.7%) followed by the Netherlands (n = 3, 12.5%) and Spain (n = 3, 12.5%). The sample size ranged from 42 to 24,735 elderly people living with HIV (median = 396). The average age ranged from 50 to 76 years and percentage of male ranged from 55.6% to 94.6%.

**Table 1:**
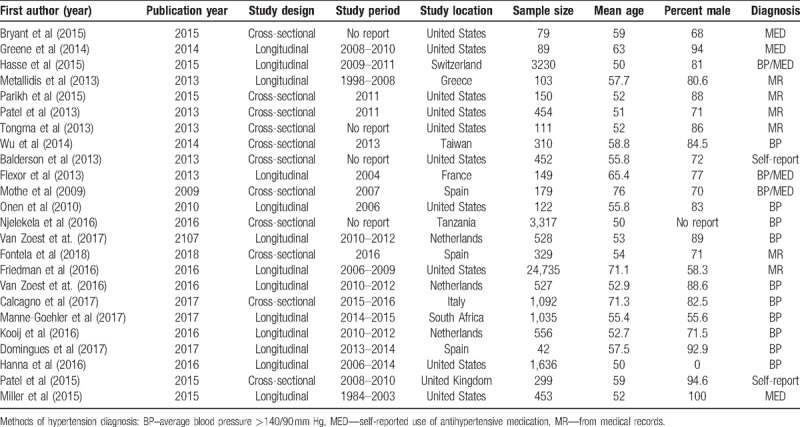
Overall characteristics of included studies.

### 3.2 Risk of bias of included studies

**Figure 1:**
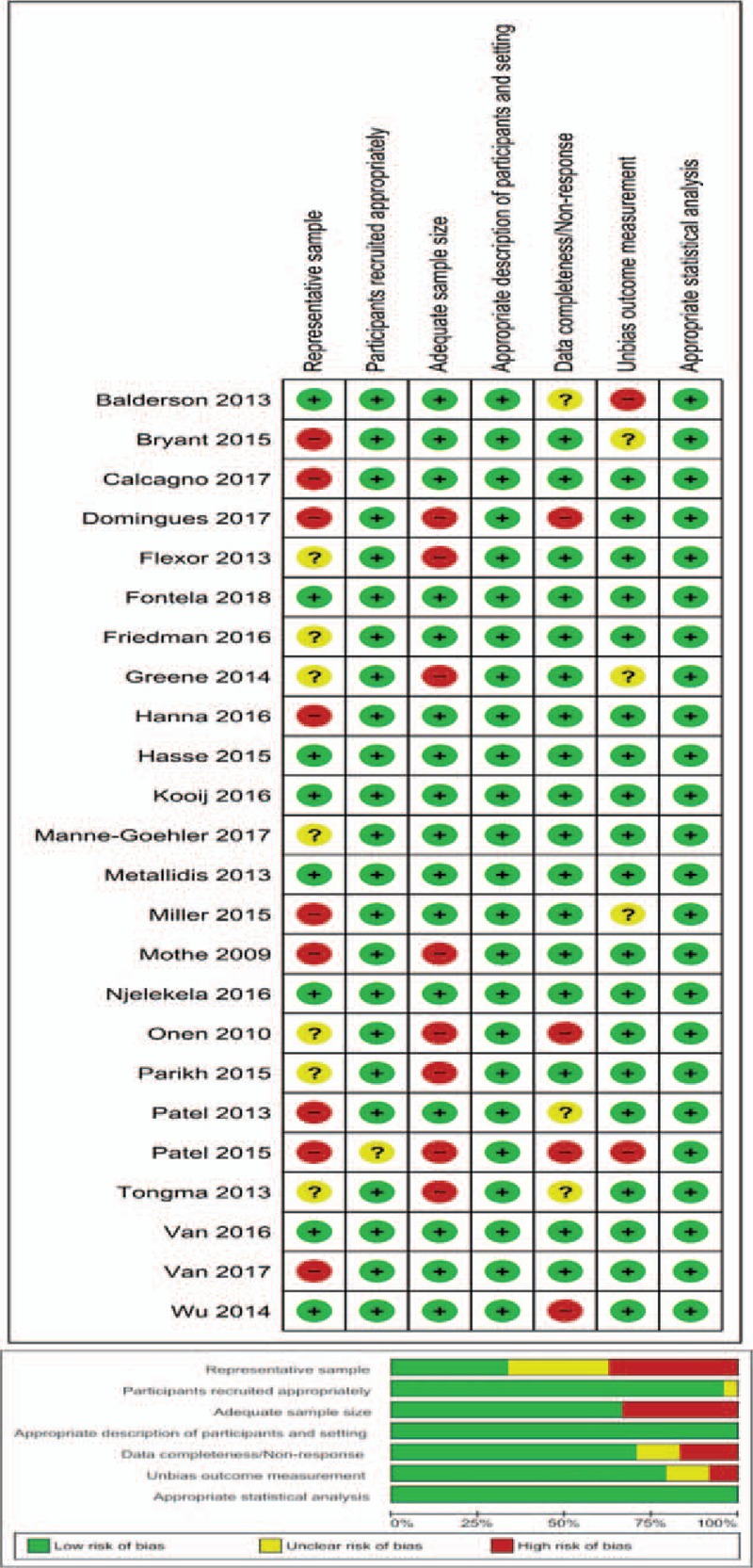
Risk of bias assessment of included studies.

The results of risk of bias assessment were presented in Figure 1. About two-thirds of the articles included in the analysis have sufficient sample size and the data completeness and respondent rate were adequate. However, only one-third of the articles could be clearly judged to have a representative sample. In almost all the articles, participants’ recruitment, description of the study participants, and data analysis were adequate. Bias was observed in the procedures for assessing outcome in two of the articles while the degree of bias could not be ascertained in three articles.

### 3.3 Prevalence of hypertension among elderly people living with HIV

**Figure 2:**
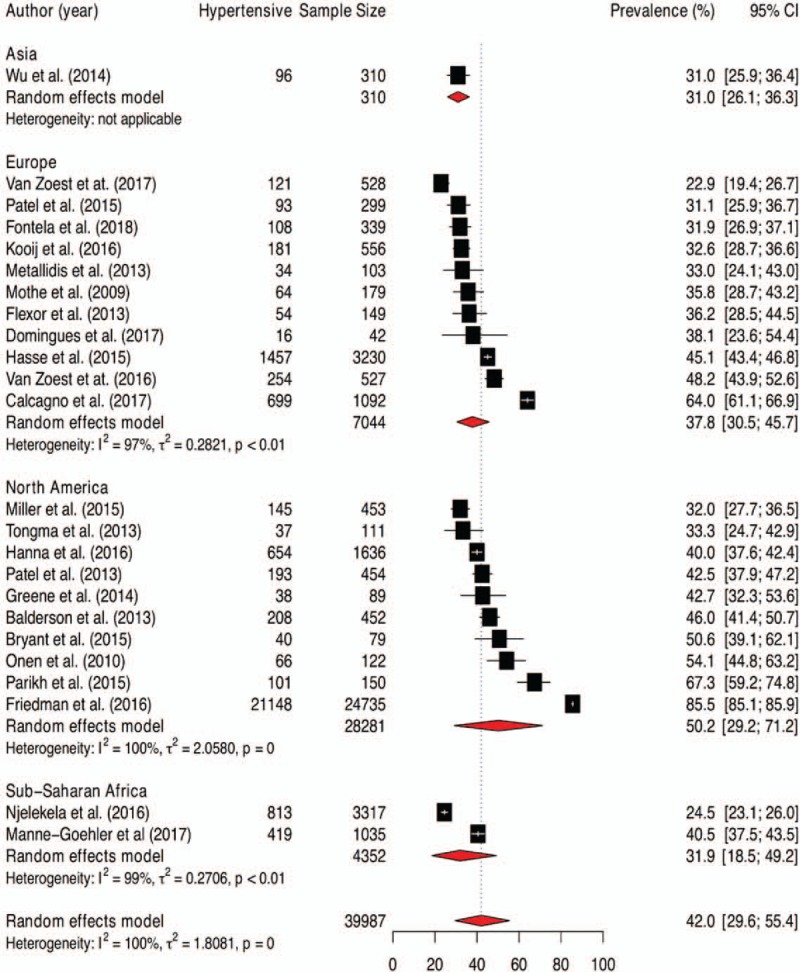
Pooled prevalence of hypertension among patients aged 50 and older living with HIV.

**Figure 3:**
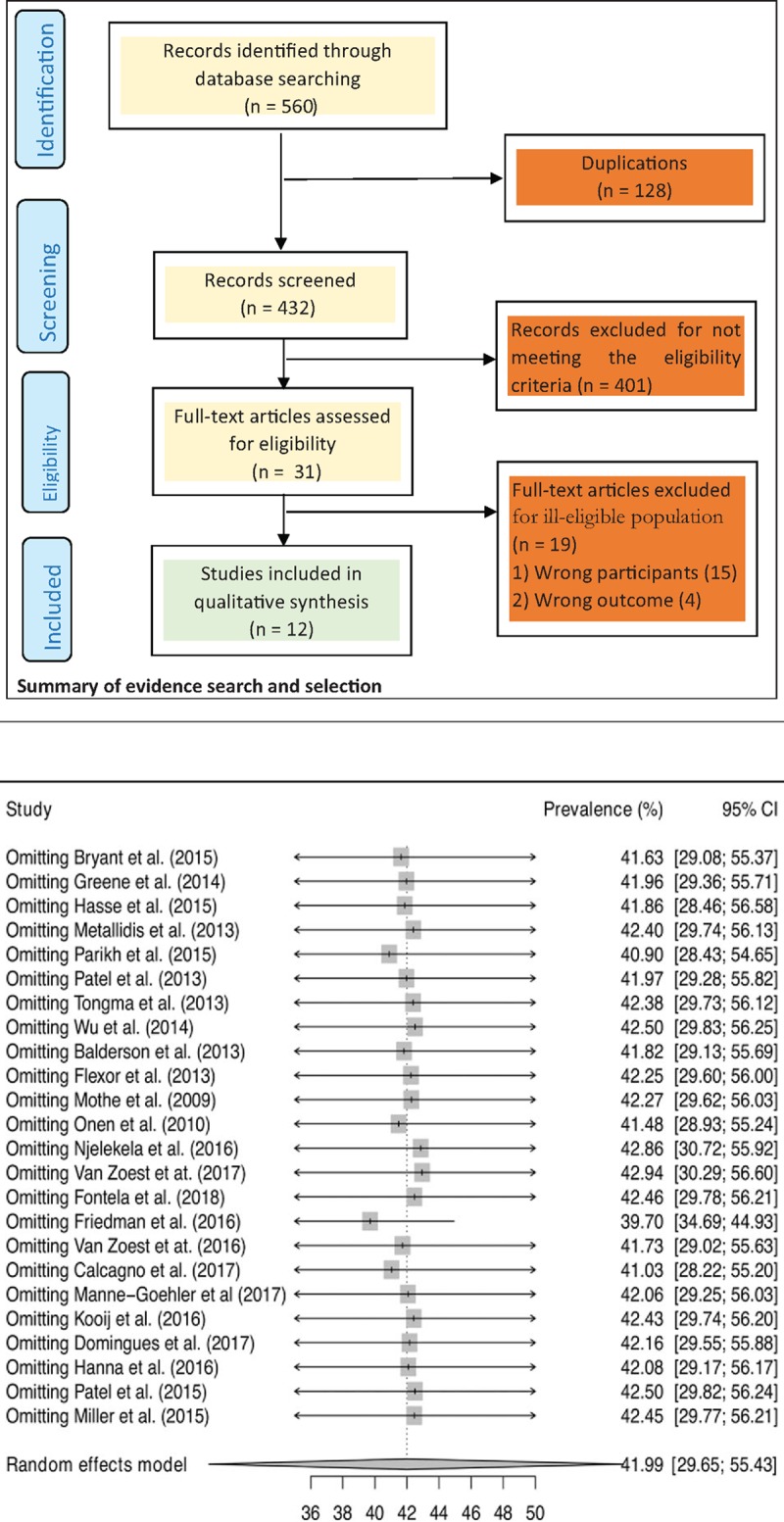
Leave-one-out sensitivity analyses.

**Figure 4:**
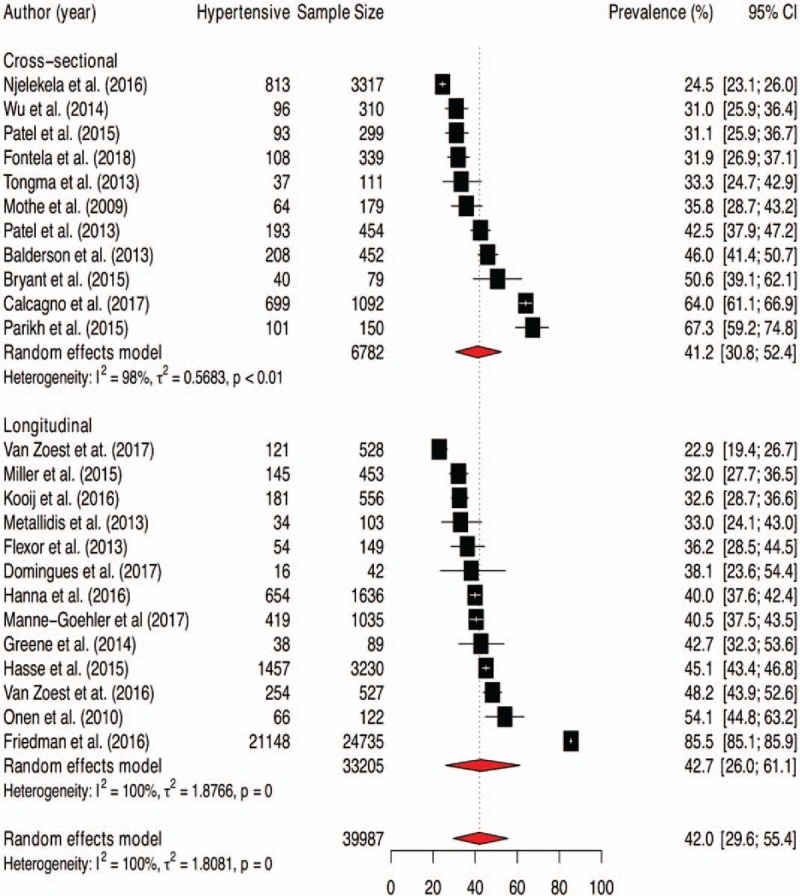
Pooled prevalence of hypertension among elderly people living with HIV by different subgroup.

The prevalence rates of hypertension among elderly people living with HIV and 95% CIs from individual studies with a pooled estimate are shown in Figure 2. The pooled prevalence of hypertension for all studies yielded an estimate of 42.0% (95% CI 29.6% to 55.4%). The *I*^*2*^ statistics was 100%, indicating statistically significant heterogeneity among the studies. The results of leave-one-study-out sensitivity analyses showed that no study had undue influence on pooled hypertension prevalence (Fig. 3). Figure 2 also show subgroup analysis by different geographical regions. The prevalence of hypertension was observed to be higher among studies conducted in North America (50.2%, 95% CI 29.2%–71.2%, 10 studies, 28281 participants) compared with studies from Europe (37.8%, 95% CI 30.5% to 45.7%, 11 studies, 7044 participants), sub-Saharan Africa (SSA) (31.9%, 95% CI 18.5%–49.2%, 2 studies, 4352 participants) and Asia (31.0%, 95% CI 26.1%–36.3%, 1 study, 310 participants). However, this difference did not reach statistically significant level (p-value for interaction = 0.312). The pooled prevalence estimates from cross-sectional studies (41.2%, 95% CI 30.8%–52.4%, 11 studies, 6782 participants) and longitudinal studies (42.7%, 95% CI 26.0–61.1, 13 studies, 33205 participants) were similar (Fig. 4).

### 3.4 Factors modifying the prevalence of hypertension as identified by meta-regression analysis

**Figure 5:**
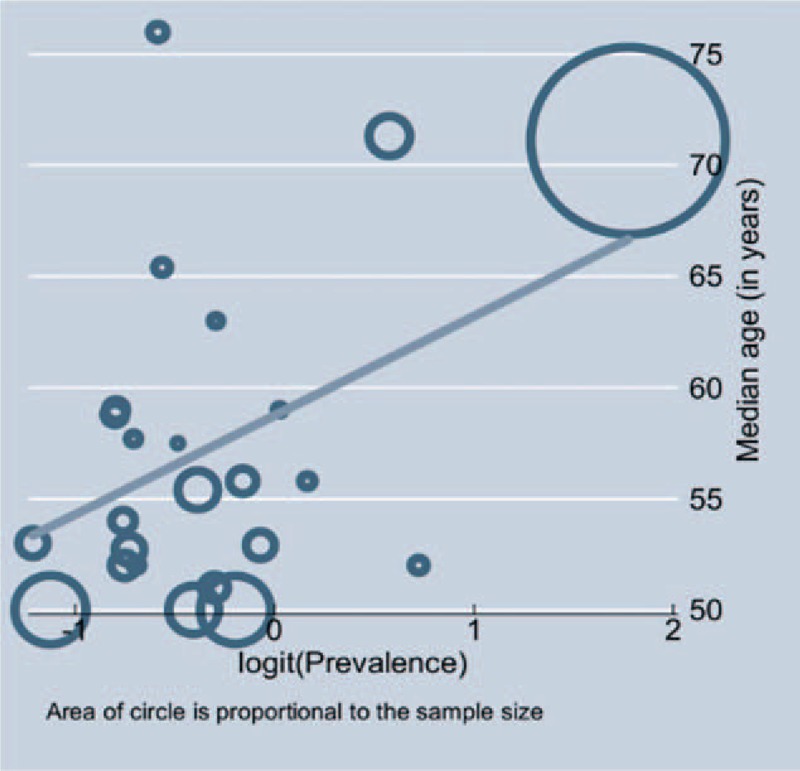
Relationship between mean age and hypertension prevalence.

Factors associated with the prevalence estimates and proportion of explained variability in the prevalence estimates as identified by meta-regression analyses are shown in Table 2. In a series of meta-regression analyses, only mean age was statistically significantly associated with the prevalence estimates; such that for every 10 years increase in the mean age of the participants, hypertension prevalence increased by 45% (OR = 1.45, 95% CI 1.01–2.09) (Table 2). In addition, Figure 5 shows the relationship between the mean age of the participants and hypertension prevalence without ignoring the size of each study population. Moreover, Figure 5 indicates that hypertension prevalence has a direct relationship with mean age of the participants.

**Table 2:**
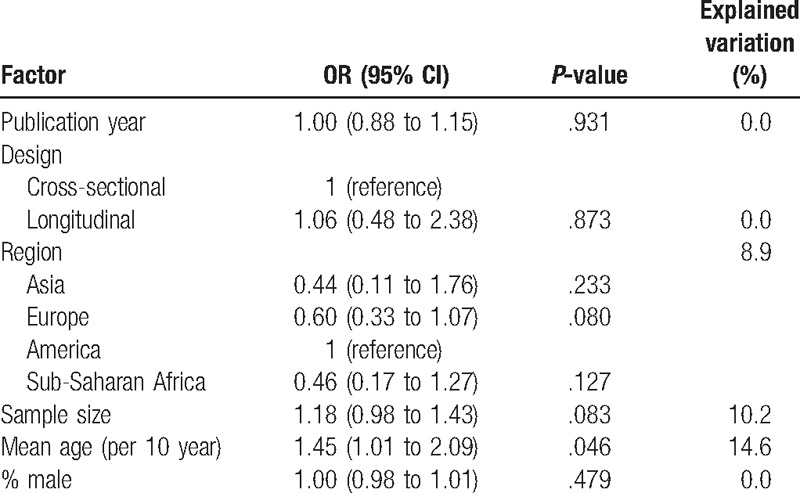
Factors associated with prevalence estimates identified by meta-regression analysis.

## 4 Discussion

To the best of our knowledge, this is the first systematic review that estimated the global prevalence of hypertension mainly in EPLWH aged 50 years and older. The underlying factors responsible for the observed differences in HIV prevalence among the study populations were examined. We observed that about 42% of EPLWH aged ≥50 years were hypertensive. This estimate is in agreement with the observation made by Xu and colleagues^[19]^; they found that the prevalence of hypertension among EPLWH aged ≥50 years was 40.3%. This indicates that out of 4.2 million EPLWH worldwide, about 2 million of them are hypertensive.^[31]^

There was variation in hypertension prevalence among the study populations. North America appear to have the highest prevalence of hypertension among EPLWH aged ≥50 years, followed by Europe, SSA, and Asia but the observed variation was not statistically significant. Among the factors considered in the meta-regression analysis to explain the observed heterogeneity in hypertension prevalence, only the mean age of the participants in each study was found to explain a considerable part of variation in prevalence of hypertension among EPLWH aged ≥50 years. This could be explained by the established relationship between age and occurrence of hypertension.^[32–34]^ However, it is important to note that the observed effect of age on the prevalence of hypertension might have partly contributed by the number of years that the participants have been exposed to HIV and ART.^[35,36]^

Given that this study involved both cross-sectional and cohort studies, we examined whether the different types of study design used have influence on the estimated prevalence of hypertension among EPLWH aged ≥50 years. Interestingly, the estimated prevalence rates of hypertension obtained from cross-sectional and cohorts were very similar. This indicates that the selected EPLWH aged ≥50 years in both cross-sectional and cohort studies yielded study populations that were similar. It is important to mention that only 2 studies were conducted in SSA among studies found to be eligible for inclusion. This may be due to inadequate research funding and HIV research capacity to answer relevant research questions in this setting^[37]^ even though the region accounts for more than two-thirds of people living with HIV globally.^[38]^

Findings from this study are consistent with previous studies that have established a positive relationship between HIV infection/ART and occurrence of hypertension. Studies have found that HIV infection can trigger atherogenesis through persistent activation of immune cell by the virus, even in those who had achieved viral suppression.^[35,39]^ Also, certain antiretroviral drugs have been implicated to cause dyslipidemia which will invariably lead to endothelia damage.^[36,40–43]^ In addition, prior studies have shown that aging on its own is a risk factor for hypertension.^[32–34]^ Thus, it was interesting that this study confirmed that aging contributed to the observed prevalence of hypertension based on the synergy between aging and HIV infection. Our observation in this study showed that 2 out 5 EPLWH are hypertensive. It indicates why it is relevant for all HIV stakeholders to integrate the management of hypertension and other prevalent co-morbidities into HIV management package.

We ensured that all eligible studies were included in the meta-analysis as our literature searches were not restricted based on language, year of publication, and geographic location. Most of the studies included were found to be of high quality, indicating low risk of bias in the prevalence rates of hypertension considered. However, this study has some limitations that is worth mentioning. Contrary to the observations in high-income countries, it may be difficult to actually estimate the actual hypertension prevalence in SSA among EPLWH as people residing in this region have a lower life expectancy. However, rectifying this limitation was beyond our control as all eligible studies identified in the literature were considered. Furthermore, it was impossible to explore heterogeneity adequately because individual-patient level characteristics were not captured in all the included studies.

In conclusion, this study estimated the global prevalence of hypertension in EPLWH. The present study will serve as a source of vital information for HIV program implementers and donors to design a comprehensive management package for HIV patients.

## Acknowledgments

The authors gratefully acknowledge the International Research Center of Excellence, Institute of Human Virology, Abuja, Nigeria for their support.

## Author contributions

Conception and design: P Dakum, G.A. Kayode, V.T. Adekanmbi, OA. Uthman. Collection and assembly of data: P Dakum, G.A. Kayode, O.A. Uthman. Analysis and interpretation of the data: P Dakum, G.A. Kayode, O.A. Uthman, V.T. Adekanmbi, A. Alash’le, Y.K. Avong, J. Okuma, E. Onyemata, T. Ali. Drafting of the article: P Dakum, G.A. Kayode, O.A. Uthman, V.T. Adekanmbi, A. Alash’le, Y.K. Avong, J. Okuma, E. Onyemata, T. Ali. Critical revision of the article for important intellectual content: P Dakum, G.A. Kayode, O.A. Uthman, V.T. Adekanmbi, A. Alash’le, Y.K. Avong, J. Okuma, E. Onyemata, T. Ali. Final approval of the article: P Dakum, G.A. Kayode, O.A. Uthman, V.T. Adekanmbi, A. Alash’le, Y.K. Avong, J. Okuma, E. Onyemata, T. Ali. Administrative, technical, or logistic support: P Dakum, G.A. Kayode, A. Alash’le

**Conceptualization:** Gbenga A Kayode, Patrick Dakum, Victor T. Adekanmbi, Olalekan A. Uthman.

**Data curation:** Gbenga A Kayode, Patrick Dakum, James Okuma, Ezenwa Onyemata, Taofeekat Ali, Olalekan A. Uthman.

**Formal analysis:** Gbenga A Kayode, Patrick Dakum, Abimiku Alash’le, Yohanna K. Avong, James Okuma, Victor T. Adekanmbi, Olalekan A. Uthman.

**Investigation:** Patrick Dakum, Abimiku Alash’le, Yohanna K. Avong.

**Methodology:** Gbenga A Kayode, Patrick Dakum, Abimiku Alash’le, Yohanna K. Avong, James Okuma, Ezenwa Onyemata, Taofeekat Ali, Victor T. Adekanmbi, Olalekan A. Uthman.

**Project administration:** Gbenga A Kayode.

**Software:** Gbenga A Kayode, Patrick Dakum, James Okuma, Victor T. Adekanmbi, Olalekan A. Uthman.

**Validation:** Gbenga A Kayode, Patrick Dakum, Abimiku Alash’le, Yohanna K. Avong, James Okuma, Ezenwa Onyemata, Taofeekat Ali, Victor T. Adekanmbi, Olalekan A. Uthman.

**Visualization:** Patrick Dakum.

**Writing – Original Draft:** Gbenga A Kayode, Patrick Dakum, Abimiku Alash’le, Yohanna K. Avong, James Okuma, Ezenwa Onyemata, Taofeekat Ali, Victor T. Adekanmbi, Olalekan A. Uthman.

**Writing – Review & Editing:** Gbenga A Kayode, Patrick Dakum, Abimiku Alash’le, Yohanna K. Avong, James Okuma, Ezenwa Onyemata, Taofeekat Ali, Victor T. Adekanmbi, Olalekan A. Uthman.
